# Distinct Serum and Tissue Markers Predict Fibrosis in Crohn’s Disease

**DOI:** 10.3390/cells15111006

**Published:** 2026-05-31

**Authors:** Ahmed Abomhya, Ronaldo Panganiban, Syed Adeel Hassan, Brandon B. Phinney, Steven McAninch, Gregory Yochum, Irina V. Pinchuk, Terrence Barrett, Ellen J. Beswick

**Affiliations:** 1 Division of Digestive Diseases, Department of Internal Medicine, University of Kentucky, Lexington, KY 40506, USA; 2 Division of Gastroenterology and Hepatology, Department of Medicine, The Pennsylvania State University, Hershey, PA 17033, USA; 3 Division of Gastroenterology and Hepatology, Department of Medicine, University of Michigan, Ann Arbor, MI 48109, USA; 4 Division of Molecular Medicine, Department of Internal Medicine, University of New Mexico Health Sciences Center, Albuquerque, NM 87131, USA; 5 Department of Surgery, College of Medicine, The Pennsylvania State University, Hershey, PA 17033, USA; 6 Department of Molecular and Precision Medicine, College of Medicine, The Pennsylvania State University, Hershey, PA 17033, USA

**Keywords:** Crohn’s Disease, fibrosis, biomarkers, Collagen 1A1, fibronectin, MMP9, Timp-1

## Abstract

Fibrosis in Crohn’s Disease (CD) occurs when there is continuous inflammation and repair mechanisms, which may lead to fibrotic strictures and ultimately intestinal surgical resection. There are limited non-invasive biomarkers for monitoring CD activity, particularly for detecting fibrosis. Thus, we set out to identify biomarkers that could be monitored for the detection and treatment of fibrosis. We used multiplex protein arrays to examine cytokines and pro-fibrotic factors in the serum of CD patients and analyzed their reliability to predict fibrosis. These markers were confirmed in tissues and the role of cytokines in upregulating fibrotic factors was examined. Collagen 1A1, Fibronectin, MMP9, and Timp-1 in patient serum were identified as strong predictors of fibrosis. IL-1β, MCP-1 and TNFα were identified as major regulators of fibrosis factors in human tissues. Overall, we identified four serum markers that are strong indicators of fibrosis and provided some novel insight into the impact of cytokines on fibrotic factors. Taken together, these findings may lead to earlier detection of fibrosis, and improved monitoring and treatment for CD patients.

## 1. Introduction

Crohn’s Disease (CD) is a chronic, relapsing inflammatory disorder of the gastrointestinal tract marked by phenotypic heterogeneity ranging from purely inflammatory to stricturing (fibrostenotic) and fistulizing behavior [1,2]. The annual direct health care costs of CD in the United States are estimated at over $8.5 billion, with lifetime per-patient costs exceeding $400,000 [3,4]. Treat-to-target guidelines incorporate biomarkers such as fecal calprotectin and C-reactive protein to monitor disease and reduce unnecessary endoscopic procedures, reinforcing the importance of developing and validating blood- and stool-based tests for objective classification of CD phenotype to guide management decisions [5,6].

Intestinal fibrosis, characterized by excessive deposition of extracellular matrix (ECM) proteins, involves a complex interplay between intestinal epithelial cells, immune cells and mesenchymal stromal cells [1]. Although it has been suggested that inflammation initiates the fibrotic process, the mechanisms associated with the repair process and fibrosis have remained elusive. Understanding these mechanisms could help identify patients at risk for developing fibrosis as well as guide development of novel therapeutic agents designed to interrupt intestinal fibrosis. Objective clinical assessment of fibrotic strictures in CD typically involves endoscopic assessments coupled with histologic evaluation of biopsy specimens and various cross-sectional imaging and ultrasonographic modalities [2]. These approaches are invasive, resource-intensive, risk-averse, have limited availability and are subject to significant interobserver variability [3,4,5]. Limitations remain with endoscopies in the assessment of CD-associated intestinal fibrosis due to limited intestinal luminal visualization. Given the transmural nature of intestinal inflammation in CD, structural changes in the bowel wall cannot always be identified by endoscopy. As such, there is a clear need for reliable and non-invasive clinical assessment of CD-associated intestinal fibrosis.

From an inflammation perspective, non-invasive biomarkers have emerged as reliable methods for detecting and monitoring CD activity. For example, both serum C-reactive protein and fecal calprotectin are used clinically to assess CD activity [6,7]. However, the utility of these markers is limited to assessing the degree of inflammation and not detecting intestinal fibrosis. There have been several previous attempts to develop novel protein biomarkers for fibrosis, with recent studies designed to identify fibrotic processes in stricturing CD. Suggested biomarkers of CD-associated fibrostenosis include miRNAs, anti-microbial antibodies, genetic markers, serum cytokines and serum extracellular matrix molecules (ECM) [8,9]. In these systematic reviews of publications of serum ECM markers, 28 molecules were identified that are linked to disease activity, but thus far these markers have only been linked to disease activity and not to fibrosis. Another study identified the value of serum cytokines to predict failure of anti-TNFα therapy, further suggesting that serum markers are a promising approach to evaluate disease activity in IBD [10]. However, more investigation is needed to identify biomarkers of fibrosis development.

In this report, we studied the comparative expression levels of several serum biomarker candidates in patients with fibrosing CD compared to patients with inflammatory CD, and patients without IBD. Further, we examined the potential of these circulating biomarkers to predict intestinal fibrosis in patients with CD. Our findings provide new insights into the utility of serum biomarkers in the non-invasive and cost-effective risk assessment of fibrotic complications in CD, with implications for personalized disease management.

## 2. Materials and Methods

### 2.1. Patient Samples

A total of 90 patients were included in this study across two independent cohorts. The first cohort consisted of sixty patients enrolled and consented in the Penn State Carlino Family Inflammatory Bowel and Colorectal Disease Biobank (IRB Protocol #PRAMSHY98-057) with serum samples available from the time of their surgeries were included in this study. Of these sixty, twenty had a diagnosis of fibrosing CD Fibrosis as confirmed by a clinical pathology report of their surgical specimens. Another 20 had primarily inflammatory CD (Active CD) with no evidence of fibrosing disease on endoscopy, histology or radiography and 20 were healthy controls. The second cohort included 30 patients from the University of Kentucky. Biopsies were collected during standard of care colonoscopies from luminal areas of perceived ongoing inflammation (active), intestinal fibrosis/strictures and inactive disease based on endoscopists’ clinical impression during intra-procedural visualization and confirmed by post-collection review of pathology reports. Example images for identification of fibrosis are shown in Figure S1. Samples were obtained from these patients and categorized as: 11 with active CD, 9 with fibrotic CD and 10 normal controls. All biopsy samples were collected with patient consent under an approved University of Kentucky Institutional Review Board protocol (#48678). Additionally, normal-appearing tissue from six de-identified CD surgical resections were obtained from The University of Kentucky Markey Cancer Center Biospecimen and Tissue Procurement Shared Resource for treatment with cytokines described below.

### 2.2. Tissue Processing

Tissue biopsy samples were divided into 4 mg pieces and incubated for 18 h in RPMI complete media containing 10% FBS and antibiotics. Supernatants were collected for multiplex analysis. Normal-appearing tissues from CD surgical resections were divided into 8 mg pieces for incubation with 20 ng/mL of recombinant cytokines (FGF2, IL-1B, IL-8, MCP-1, and TNFα purchased from VWR, Radnor, PA, USA) to examine production of fibrosis markers in supernatants after 18 h in RPMI complete media.

### 2.3. Multiplex Arrays

Serum samples and tissue supernatants were examined by multiplex bead array for custom fibrosis marker panel (R&D Systems, Minneapolis, MN, USA) including pro-collagen 1A1, FAP, Fibronectin, and TNC. MMP9 and Timp1 were run by a Procarta custom array from ThermoFisher Scientific (Carlsbad, CA, USA) and cytokines by a Human Cytokine/Chemokine panel A 38-plex (MilliporeSigma, Burlington, MA, USA). All assays were run according to manufacturer’s instructions on a Luminex LX200 instrument (Luminex Corporation, Austin, TX, USA).

### 2.4. Primary Cultures of Fibroblasts and Cytokine Treatment

Fresh colonic human mucosal tissues were derived from non-inflamed and non-fibrotic area of discarded full-thickness surgical resection material of CD patients that were obtained from Carlino Family Colorectal Disease Biobank in compliance with IRB-approved protocol by the Pennsylvania State University. CD-derived fibroblasts (CD-Fib) were isolated according to the protocol of Mahida et al. [11]. Studies were performed with primary CD-Fib isolates at passages 4–9. Cells were cultured at 37 °C in 5% CO_2_ in complete Modified Eagle Medium (MEM) containing 10% FBS as previously described [12]. CD-Fib were treated with cytokine when reaching 70–80% of confluency with 10 ng/mL of TNF (R&D Systems), IL-1β (R&D Systems, Minneapolis, MN, USA) or FGF2 (FUJIFILM Biosciences, Santa Ana, CA, USA) for 24 h, then cells were harvested and RNA was isolated from cells as described below.

### 2.5. Quantitative RT-PCR

Total RNA was isolated using Qiagen RNAeasy mini isolation kits according to the manufacturer’s procedure (Qiagen, Germantown, MD, USA). RNA analysis was performed according to the two-step RT real-time PCR protocol as previously described [13].

### 2.6. Statistical Analysis

All statistical analyses were performed using GraphPad Prism 10 software. Results were presented as differences between means evaluated by one-way ANOVA for multiple comparisons. The Kruskal–Wallis test was used to test for an overall difference in median levels of cytokines between groups while using the Sidak adjustment for multiple testing. Associations between fibrosis factors and cytokines were analyzed using spearman correlations and prediction of fibrosis calculated by Area Unver the Curve (AUC) from Receiver Operating Characteristic (ROC) curves where >0.5 was considered significant. Values of *p* < 0.05 were considered statistically significant.

## 3. Results

### 3.1. CD Serum Samples from Patients with Fibrosis Have Increased Levels of Specific Fibrosis Markers

To investigate serum fibrosis markers in CD, a custom multiplex array was designed to quantify target proteins using bead assays. Serum samples obtained from CD patients with active disease and fibrosis were compared to healthy control samples. Patients were classified as “Active CD” if their clinical gross and microscopic pathology revealed active inflammation without fibrosis. On the other hand, patients were classified as “CD Fibrosis” if their gross and microscopic pathology report revealed evidence of active disease with fibrosis. A summary of the patients’ clinical characteristics and demographics for serum studies are shown in Table S1. Furthermore, example colonoscopy images for identification of fibrosis are shown in Figure S1.

**Figure 1:**
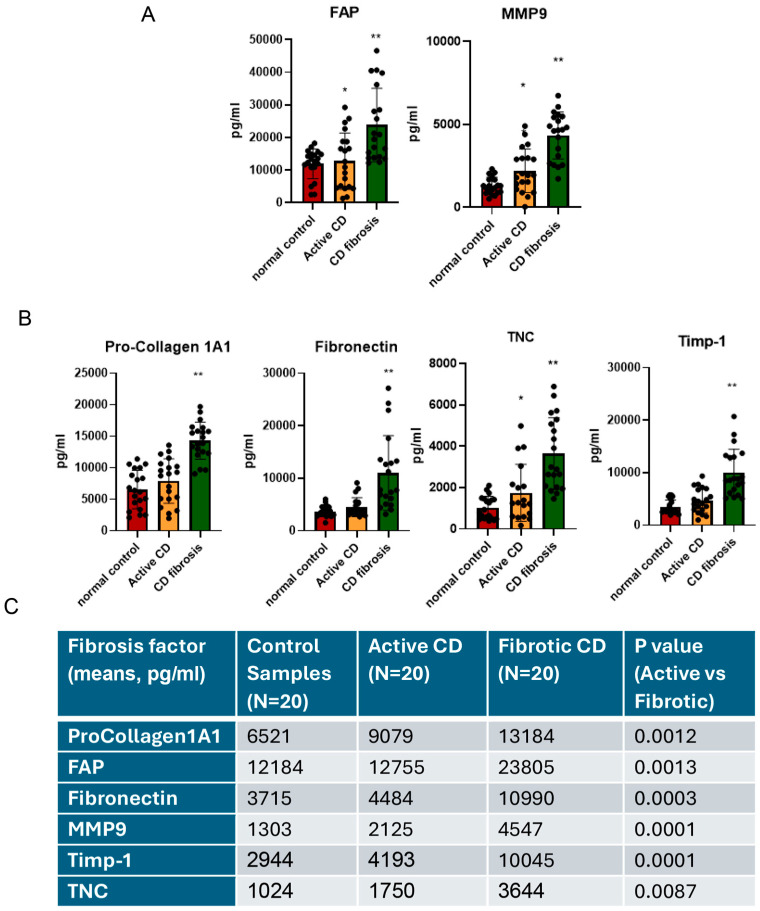
Serum fibrotic markers are increased in the serum of patients with fibrosis compared to the serum of normal patients and CD patients with active disease when analyzed by multiplex array indicating (**A**) markers significantly increased in active disease, which are further increased in fibrotic disease, (**B**) markers significantly increased in only fibrotic disease and (**C**) *p* values are shown comparing fibrotic disease to active disease. N = 20, * *p* < 0.05 compared to normal control and ** *p* < 0.05 compared to active CD.

Fibroblast-activating protein (FAP) and MMP9 were significantly increased in active CD patient serum compared to healthy control and further significantly increased in CD fibrosis serum compared to active CD serum (Figure 1A). Furthermore, Pro-Collagen 1A1, Fibronectin, Tenascin C (TNC) and Timp-1 were all found to be significantly increased in serum from CD patients with active disease and fibrosis compared to patients with active disease alone (Figure 1B). Interestingly, the markers were not significantly increased in active disease samples compared to healthy controls. The mean amounts of these serum factors are shown indicating that all of these markers are higher in CD fibrosis samples (Figure 1C). These results are consistent with the notion that pro-Collagen 1A1, Fibronectin, TNC, and Timp-1 are clinically relevant biomarkers useful in predicating and monitoring disease severity and fibrosis in CD patients.

### 3.2. CD Serum Samples from Patients with Fibrosis Have Increased Levels of Specific Cytokines

**Figure 2:**
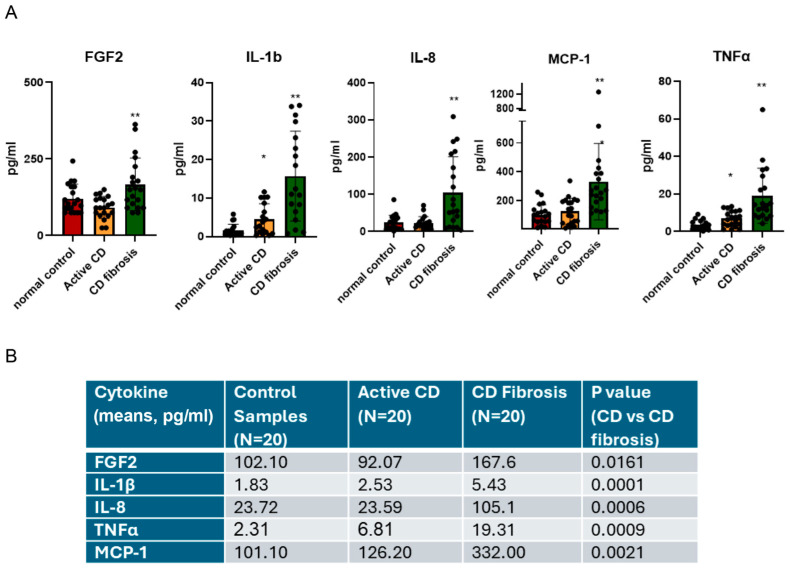
Serum cytokines are increased in the serum of patients with fibrosis compared to the serum of CD patients with active disease when analyzed by multiplex array indicating (**A**) cytokines significantly increased in fibrotic disease and (**B**) *p* values are shown comparing fibrotic disease to active disease. N = 20, * *p* < 0.05 compared to normal control and ** *p* < 0.05 compared to active CD.

Chronic inflammation has long been associated with fibrosis, but the specific mechanisms implicated in fibrosis are not well understood. In these serum samples, 7 cytokines/chemokines were found to be significantly increased in active CD with fibrosis patient serum compared to active CD serum (Figure 2), which included FGF2, IL-1β, IL-8, MCP-1, and TNFα. In contrast, cytokines/chemokines that were detectable, but not significantly increased in active CD compared to controls included FGF2, IL-8, and MCP-1. Furthermore, IL-6, MCP-3, CXCL1 and GM-CSF were increased in all CD patients, but were not significantly different between CD patients with inflammation and those with fibrosis. Figure 2B indicates mean amounts in serum for each group and *p* values for cytokines that were significantly increased in the serum of CD patients with fibrotic disease. Overall, these data suggest that serum inflammatory cytokine levels were higher when associated with fibrogenic CD compared to inflamed but not fibrotic CD. One potential interpretation from these data is that fibrotic CD has more severe inflammation (reflected by higher serum cytokine levels) and that more severe mucosal inflammation portends greater intestinal fibrogenesis.

### 3.3. Serum Fibrosis Markers Predict Fibrosis

**Figure 3:**
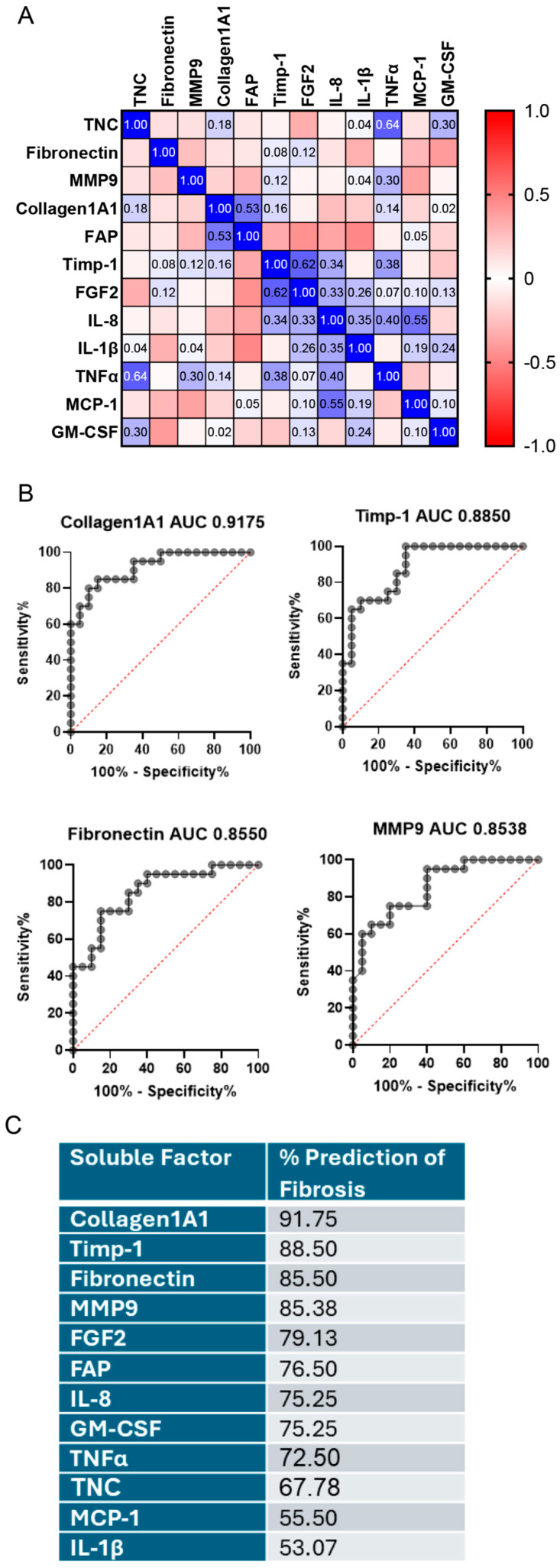
Serum fibrotic markers predict fibrosis as shown by (**A**) correlation matrix, (**B**) ROC curves with AUC and (**C**) prediction of fibrosis.

To further investigate the relationship of fibrosis-associated biomarkers and cytokines/chemokines in patient serum samples, Spearman correlations were examined. A correlation matrix was established using Spearman’s correlations in Figure 3A, which indicates in blue a number of strong correlations between cytokines/chemokines and fibrosis biomarkers with the strongest associations including Timp-1 and Fibroblast Growth Factor 2 (FGF2), followed by TNC and TNF-α, IL-8 and MCP-1 and MMP-9. To analyze the potential for these factors to predict fibrosis, ROC curves were established and the Area Under the Curve (AUC) calculated (Figure 3B). The default cutoff for ROC curve prediction is 0.5, so we report the factors with strong associations. These calculations indicated that Pro-Collagen 1A1, Fibronectin, Timp-1 and MMP9 were strong predictors of fibrosis at 91.75%, 88.50%, 85.50%, and 85.38%, respectively, Figure 3C. FGF2 also strongly predicted fibrosis at 79.13%. Taken together, our data suggest that these factors were predictive of increased mucosal fibrosis.

### 3.4. CD Tissues Express Specific Fibrosis Markers and Cytokines

**Figure 4:**
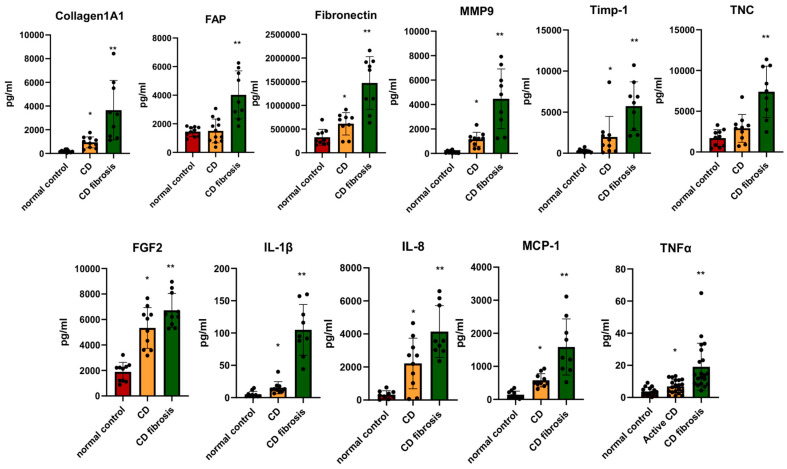
Soluble factors detected in serum are present in human tissues and increased in CD with fibrosis. N = 10, * *p* < 0.05 compared to control, ** *p* < 0.05 in CD fibrosis compared to active disease.

In order to support the serum data, specific factors in tissue samples were also examined (Table S2). Tissue supernatants from 8 mg pieces of tissue incubated in RPMI complete media for 18 h were analyzed for fibrosis factors and cytokines by multiplex arrays. Results were similar to serum data indicating that both fibrosis and cytokines are generally increased in CD, but to significantly higher levels in fibrotic patients. To note, active CD patient tissues did not have significantly increased FAP or Timp-1 compared to normal healthy control patient tissues (Figure 4). However, all markers were significantly increased in fibrotic CD tissue samples compared to active inflammatory (non-fibrotic) CD tissue samples, suggesting that both serum and tissue markers are significantly higher in active fibrotic CD compared to active inflammatory CD.

### 3.5. Cytokines Upregulate Fibrosis Markers in Human Tissues and Fibroblasts

**Figure 5:**
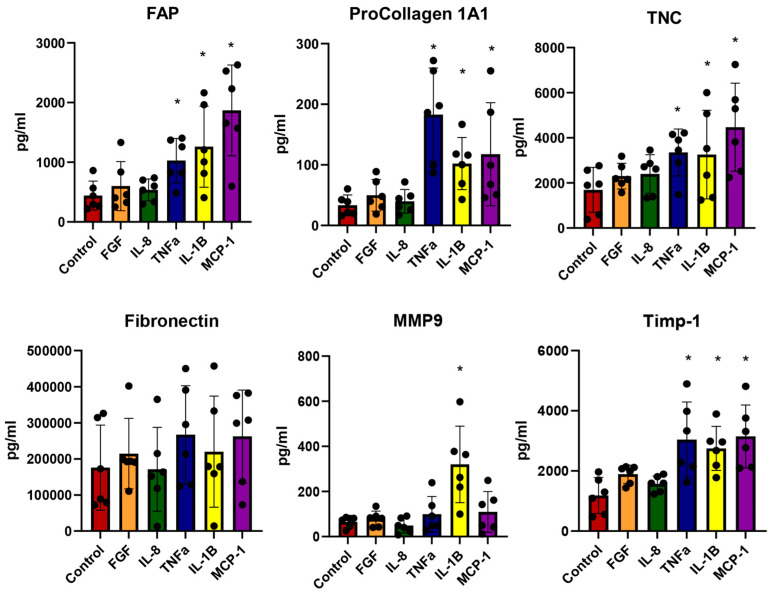
Recombinant cytokines increase fibrotic factor production in normal tissue from CD patients after 8 mg tissue pieces were incubated with 20 ng/mL of each cytokine. N = 6, * *p* < 0.05 compared to control.

Since chronic inflammation and fibrosis have long been linked, but these processes are still missing some mechanistic associations, we examined the impact of recombinant cytokines on production of fibrotic factors by colon tissues. For these studies, normal-appearing tissues from de-identified surgical resections from CD patients were utilized due to the amount of tissue required for comparisons. Tissue pieces (8 mg) were incubated with FGF2, IL-8, TNFα, IL-1β, and MCP-1 as cytokines found to be significantly increased in fibrotic tissues. After 18 h incubations, sample supernatants were examined for the production of fibrotic factors. Interestingly, we found that TNFα, IL-1β, and MCP-1 were able to increase production of FAP, ProCollagen 1A1, TNC, and Timp-1 by these tissues (Figure 5). Only IL-1β upregulated MMP-9, whereas none of these cytokines increased fibronectin.

**Figure 6:**
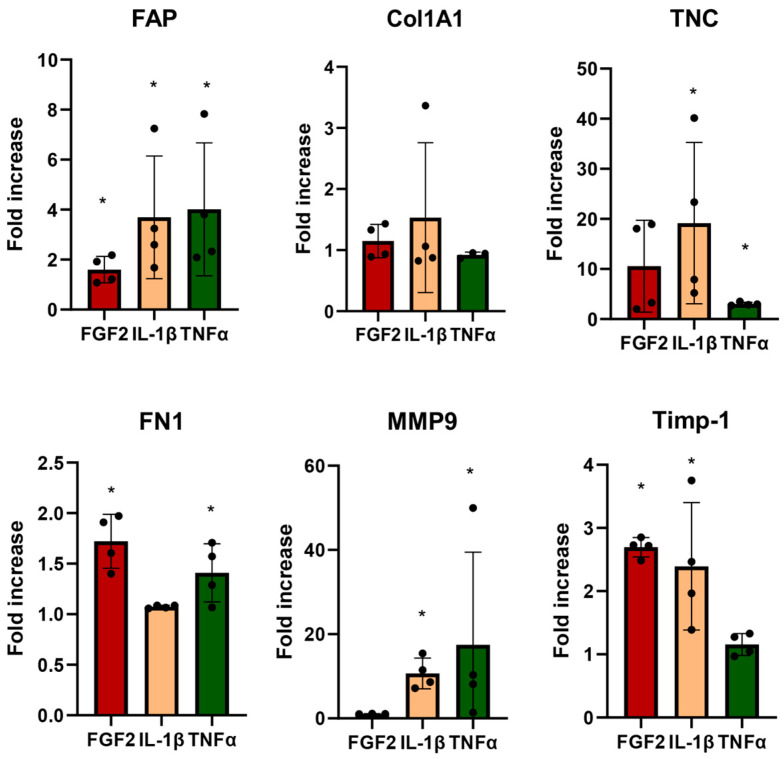
Primary culture of fibroblasts derived from non-inflamed intestinal areas of CD patients were treated with 10 ng/mL of recombinant TNF, IL1b or FGF2 for 24 h, and analyzed by qRT-PCR. N = 4, * *p* < 0.05.

Because fibroblasts play a central role in fibrotic tissue remodeling, as demonstrated by our group and others [14,15], we investigated whether stimulation with candidate cytokines induces the expression of key pro-fibrotic mediators identified in serum and elevated in CD tissue. When cultured fibroblasts (CD-Fibs) from non-inflamed area of CD patient tissues were treated with TNFα or IL-1β for 24 h, a significant increase in gene expression of *MMP9*, *TNC*, and *FAP* was observed, while exposure to FGF2 increased the expression of *FAP*, but not *MMP9* and *TNC* (Figure 6). Furthermore, treatment of CD-Fib with IL-1β or FGF2, but not TNFα resulted in the significant increase in *TIMP1*. The expression of *Fn1* by CD-Fibs was only slightly increased by TNFα and FGF2, but not IL1β. Interestingly, none of the tested cytokines directly impacted expression of *Col1A1* in CD-Fib (Figure 6). These observations suggest that the significant impact of TNFα on ProCollagen 1A1 production observed with CD-Tissue explant (Figure 5) is likely to involve additional cellular players and molecules to induce its expression within fibroblasts. Overall, these data indicate a direct and somewhat overlapping but not fully redundant role for TNFα, IL1β and FGF2 s in regulating fibrotic factors. Further studies are required to determine the mechanisms of fibronectin and collagen upregulation in fibroblasts in CD.

## 4. Discussion

Serum biomarkers are a potentially valuable, non-invasive, and inexpensive way to monitor disease status. To date, very few serum biomarkers exist for IBD. One serum marker previously linked to the severity of mucosal inflammation is CRP, particularly in CD [16]. Previous studies have shown that changes in CRP illustrate how serum marker monitoring with CRP levels provides a useful tool for monitoring patient disease state. Since nearly half of CD patients undergo intestinal resection within 10 years of diagnosis [17,18], new biomarkers could be useful in early detection of fibrosis. Discovering a reliable serum biomarker signature that differentiates between active CD without fibrosis and CD with fibrosis can lead to innovative and less invasive approaches to manage CD patients at risk for developing fibrostenotic complications.

To approach more in-depth serum analysis for CD patients, we designed a multiplex panel to examine circulating markers in CD patients. The markers in this serum panel were compared between serum samples from active CD without fibrosis and active CD with fibrosis. We found that FAP was elevated in active CD patient serum and rose further in fibrotic active CD patient serum. FAP has recently gained attention for its role in fibrotic lesions implicated in cross-talk between fibroblasts and monocytes, a major regulator of cellular inflammatory responses. Two independent reports characterized a TWIST1^+^FAP^+^ fibroblast subset enriched in fibrotic intestinal lesions that sustains fibrostenosis [19,20]. These findings provide a mechanistic bridge from tissue-resident fibrogenic fibroblasts to the circulating ECM/cytokine milieu we measured, and they emphasize FAP as both a marker and a functional driver of intestinal fibrosis. One prospective study reported reduced circulating FAP (cFAP) in IBD patients compared with controls and suggested cFAP correlated with endoscopic mucosal healing [20], whereas immunohistochemical and functional ex vivo data show increased tissue FAP in strictures and reduced fibrogenic output after FAP inhibition [20,21]. In contrast, our findings show that FAP may be upregulated in serum and tissue in patients with fibrosis; however, other markers were stronger predictors of fibrosis than FAP.

Perhaps more valuable as serum markers linked with fibrosis are Pro-Collagen 1A1, Fibronectin, MMP9, and Timp-1 because these markers were not found to be significantly increased in the serum samples of CD patients with active non-fibrotic disease, whereas levels were significantly increased in patients with active disease and fibrosis. Our finding that Pro-Collagen 1A1 (a type I collagen formation marker) strongly discriminates fibrotic CD is consistent with a net shift toward type I collagen deposition in stricturing lesions and supports using both formation and degradation assays to better define active fibrogenesis [22]. Fibronectin and the MMP9/TIMP axis further corroborate an ECM deposition program in fibrostenosis. We also found MMP9 and Timp-1 to be elevated in the serum of active CD patients, which was significantly higher in fibrotic CD patients. MMPs are complex in nature, but we have routinely found MMP9 to be elevated in CD [23,24]. Other studies suggest a similar phenomenon that MMP9 may be involved in barrier disruption in IBD [25], and another also suggests pro-MMP9 as a marker of fibrosis in IBD [26]. An imbalance favoring TIMP-1 alongside increased collagen formation and matrix glycoprotein deposition is a biologically plausible route to net ECM accumulation and stricture development [27,28]. Historical literature on plasma fibronectin is nuanced. Lower levels have been reported in severe inflammation, yet associations between higher fibronectin and subsequent stricture formation were reported [29]. These findings highlight how chronic inflammation, tissue sequestration, and chronic ECM deposition can produce disparate plasma signals that require contextual interpretation [30]. The simultaneous increase in fibronectin, MMP-9, TIMP-1 and ProCollagen1A1 in our fibrotic cohort maps well for these mechanisms. Overall, our serum profiling demonstrates a coherent, multi-analyte signature that distinguishes histologically confirmed fibrostenotic CD from active inflammatory disease and from healthy controls.

We also examined cytokine markers given the known link between inflammation and fibrosis. In serum, FGF2, IL-1β, IL-8, MCP-1, and TNFα are cytokines that were significantly increased between active CD and fibrotic active CD patient samples, suggesting their potential role in promoting fibrotic responses. FGF2 showed a 75% prediction for fibrosis using AUC analysis of ROC curve, while the other cytokines were in the 50–60% range. However, the strongest prediction of fibrosis from serum markers were Pro-collagen 1A1, Fibronectin, MMP9, and Timp-1, suggesting these markers could be used in a serum panel to predict fibrosis. To support serum data for potential biomarker analysis, the fibrosis markers and cytokines were examined in tissue samples where these markers were similarly increased in fibrotic tissues over inflamed tissues.

There has long been suggested a link between inflammation and fibrosis, but the mechanisms are not yet clear. To investigate this, we treated normal-appearing tissues from a set of CD patient surgical resections with recombinant cytokines, where we found IL-1β, TNF-α, and MCP-1 to be the major regulators of tissue production of fibrotic markers FAP, ProCollagen 1A1, Timp-1, TNC and MMP9. Furthermore, IL-1β and TNFα and to a lesser extent FGF2 also upregulated expression of several of these markers on cultured fibroblasts from CD patients, suggesting a direct mechanism linking inflammatory cytokines to fibrosis. These studies are supported by others indicating that selected cytokines regulate fibrotic factors [31,32].

There are several caveats to these studies such as medications, particularly biologics, which may affect production of these inflammatory and fibrotic factors. However, there were significant changes between active CD and fibrotic CD in both serum and tissues. Although our panel performed well in ROC analysis, adequate external validation, including prospective cohorts with paired histology is needed to further test our findings and detailed associations with patient history, medications and other medical diagnoses that may affect inflammation and fibrotic factor analysis. In general, larger studies may support ranges of cytokines and fibrotic factors in fibrotic disease that may indicate fibrosis and perhaps support a non-invasive test for fibrotic disease.

In conclusion, integrating serum ECM formation markers, matrix glycoproteins, protease inhibitor balance, and selected myeloid cytokines yields a biologically consistent and diagnostically promising signature of fibrostenotic Crohn’s Disease. Fecal calprotectin and CRP are useful inflammatory markers, but do not reliably indicate a fibrostenotic phenotype [8,33]. Our study may serve as an initial scientific basis for future investigations exploring the potential of Pro-Collagen 1A1, Fibronectin, MMP9, and Timp-1 as biomarkers for predicting fibrotic complications in patients with CD. A validated, standardized serum panel could therefore be used for risk stratification, surveillance, and selection of patients for anti-fibrotic trials. Here, we identified a novel distinct serum biomarker panel that could distinguish inflamed fibrotic CD from inflammatory non-fibrotic CD. This is the first time a process to differentiate CD phenotypes has been established. We also showed that fibrotic CD phenotypes have a distinct inflammatory cytokine signature. Our results support prospective validation of a multiplex serum panel for early identification of patients at risk for fibrosis, with the ultimate aim of enabling earlier, mechanism-directed interventions and reducing dependence on invasive investigations.

## Data Availability

The original contributions presented in this study are included in the article/Supplementary Materials. Further inquiries can be directed to the corresponding author.

## Supplementary Materials

The following supporting information can be downloaded at: https://www.mdpi.com/article/10.3390/cells15111006/s1, Figure S1. Example images of fibrosis and stricture; Table S1. Demographics and Clinical Characteristics of Patients Serum Study (Penn State); Table S2. Demographics and Clinical Characteristics of UK cohort.

## Abbreviations

The following abbreviations are used in this manuscript:

CD	Crohn’s Disease
ECM	Extracellular matrix
CD-Fib	Crohn’s Disease-derived fibroblasts
FAP	Fibroblast-activating protein
TNC	Tenascin C
AUC	Area under the curve
ROC	Receiver Operating Characteristic
