# Supplementary material for: Distinct Serum and Tissue Markers Predict Fibrosis in Crohn’s Disease
Source: Cells. 2026 May 31;15(11):1006. doi: 10.3390/cells15111006 (PMC13256602; doi:10.3390/cells15111006)
Supplement: Supplementary file 1 [file cells-15-01006-s001.zip › cells-4311937-supplementary.pdf]

Supplementary Data:

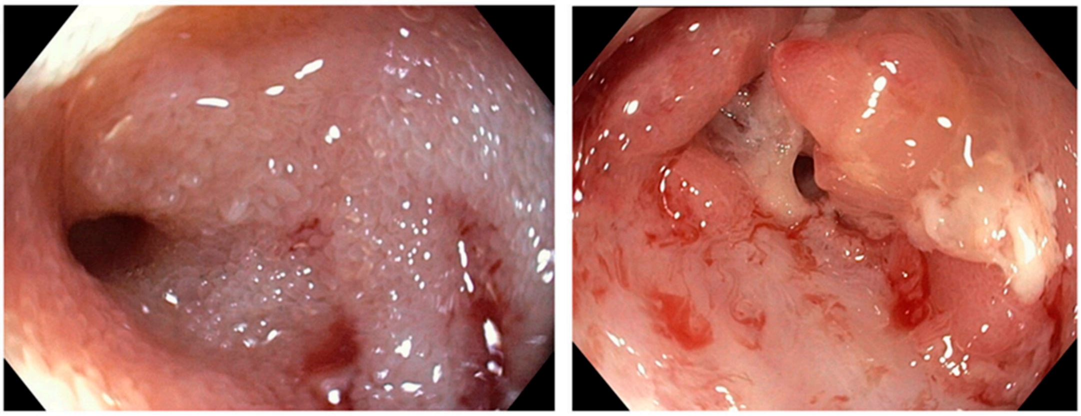

Figure S1. Example images of fibrosis and stricture.

Table S1. Demographics and Clinical Characteristics of Patients Serum Study (Penn State).

	Active CD (n = 20)	CD Fibrosis (n = 20)	Normal Control (n = 20)
**Gender = Male, n(%)**	6 (30.0)	16 (80.0)	10 (50.0)
**Race, n(%)**			
Black, Not Hispanic	1 (5.0)	0 (0.0)	0 (0.0)
White, Hispanic	1 (5.0)	1 (5.0)	0 (0.0)
White, Not Hispanic	18 (90.0)	19 (95.0)	20 (100.0)
**Smoking Status, n(%)**			
Current	7 (35.0)	5 (25.0)	3 (15.0)
Former	6 (30.0)	5 (25.0)	7 (35.0)
Never	7 (35.0)	10 (50.0)	9 (45.0)
NA	0 (0.0)	0 (0.0)	1 (5.0)
**NSAID Use, n(%)**			
No	9 (45.0)	10 (50.0)	9 (45.0)
Yes	11 (55.0)	9 (45.0)	11 (55.0)
NA	0 (0.0)	1 (5.0)	0 (0.0)

	Active CD (n = 20)	CD Fibrosis (n = 20)
**IBD Duration, mean (SD)**	21.16 (8.76)	20.86 (8.84)
**Montreal Classification: Age at Diagnosis, n (%)**		
A1: < 16 yrs	3 (15.0)	2 (10.0)
A2: between 17 and 40 yrs	15 (75.0)	16 (80.0)
A3: > 40 yrs	2 (10.0)	2 (10.0)
**Montreal Classification: Disease Behavior n (%)**		
B1: Non-stricturing, non-penetrating	4 (20.0)	1 (5.0)
B2: Stricturing	5 (25.0)	7 (35.0)
B2B3: Stricturing and Penetrating	6 (30.0)	9 (45.0)
B3: Penetrating	5 (25.0)	3 (15.0)
**Montreal Classification: Disease Location, n (%)**		
L1: Ileal	3 (15.0)	7 (35.0)

L2: Colonic	1 (5.0)	1 (5.0)
L3: Ileocolonic	16 (80.0)	12 (60.0)
**Montreal Classification: Perianal Involvement = Yes, n(%)**	11 (55.0)	5 (25.0)
**Steroid Use = Yes, n(%)**	16 (80.0)	16 (80.0)
**IBD-specific Therapies, n(%)**		
6MP	1 (5.6)	0 (0.0)
Azathioprine	4 (22.2)	5 (25.0)
Anti-Integrin	1 (5.6)	0 (0.0)
Mesalamine	2 (11.1)	3 (15.0)
None	1 (5.6)	0 (0.0)
Anti-TNF	9 (50.0)	12 (60.0)

Table S2. Demographics and Clinical Characteristics of UK cohort.

	Active CD (n =11)	CD Fibrosis (n = 11)	Normal Control (n = 10)
**Gender = Male, n(%)**	7 (63.63)	5 (45.45)	5 (50)
**Race, n(%)**			
Black, Not Hispanic	1 (9.1)	0 (0)	0 (0)
White, Hispanic	0 (0)	0 (0)	1 (10)
White, Not Hispanic	10 (90.9)	11 (100)	9 (90)
**Smoking Status, n(%)**			
Current	1 (9.1)	1 (9.1)	0 (0)
Former	2 (18.2)	4 (36.4)	4 (40)
Never	8 (72.7)	6 (54.5)	6 (60)
NA	0 (0)	0 (0)	0 (0)
**NSAID Use, n(%)**			
No	8 (72.7)	7 (63.6)	8 (80)
Yes	3 (27.3)	4 (36.4)	2 (20)
NA	0 (0)	0 (0)	0 (0)

	Active CD (n = 11)	CD Fibrosis (n = 11)
**IBD Duration, mean (SD)**	10.7 (8.55)	13.54 (13.98)
**Montreal Classification: Age at Diagnosis, n (%)**		
A1: < 16 yrs	3	0
A2: between 17 and 40 yrs	7	7
A3: > 40 yrs	1	4
**Montreal Classification: Disease Behavior n (%)**		
B1: Non-stricturing, non-penetrating	3	3
B2: Stricturing	4	2
B2B3: Stricturing and Penetrating	3	4
B3: Penetrating	1	2
**Montreal Classification: Disease Location, n (%)**		
L1: Ileal	4	2
L2: Colonic	3	0
L3: Ileocolonic	4	9

**Montreal Classification: Perianal Involvement = Yes, n(%)**	4	6
**Steroid Use = Yes, n(%)**	8	11
**IBD-specific Therapies, n(%)**		
6MP	0	0
Azathioprine	0	0
JAK inhibitor	3	1
IL23 inhibitor	2	3
Anti-Integrin	1	2
Mesalamine	0	0
None	4	2
Anti-TNF	1	3
